# Draft Genome Sequences of Escherichia coli and Enterococcus faecalis Coisolated from Polymicrobial Extraintestinal Infections of Chickens and Turkeys

**DOI:** 10.1128/mra.01163-22

**Published:** 2023-02-21

**Authors:** Grayson K. Walker, Lyndy Harden, M. Mitsu Suyemoto, Siddhartha Thakur, Megan Jacob, Luke B. Borst

**Affiliations:** a Department of Population Health and Pathobiology, College of Veterinary Medicine, North Carolina State University, Raleigh, North Carolina, USA; University of Maryland School of Medicine

## Abstract

Coinfections by avian pathogenic Escherichia coli (APEC) and Enterococcus faecalis in poultry with colisepticemia have become increasingly recognized. Here, we report draft genome sequences of 18 APEC and 18 E. faecalis strains coisolated from lesions of diseased poultry.

## ANNOUNCEMENT

Avian pathogenic Escherichia coli (APEC) and Enterococcus faecalis cause septicemic disease in poultry resulting in substantial mortality, economic burdens, and public health risks (1, 2). Interestingly, interactions between these two pathogens in polymicrobial extraintestinal infections can augment host disease severity (3). Although APEC isolates from poultry have been sequenced, coisolated APEC and E. faecalis from polymicrobial infections of poultry have received less attention despite their clinical and epidemiological significance.

We previously described 31 APEC and 31 E. faecalis strains coisolated from systemic infections of poultry from 2016 to 2019 at the North Carolina State University Veterinary Teaching Hospital in Raleigh, North Carolina, and found that APEC exhibited growth augmentation in mixed culture with E. faecalis coisolates (2). All APEC and E. faecalis coisolates were collected with Stuart swabs (BD; 220144) from lesions identified grossly as omphalitis or splenomegaly. Samples were incubated aerobically at 37°C on Trypticase soy agar with 5% sheep blood for 18 to 24 h, and isolates from cultures that contained a mixed growth of only Escherichia coli and *Enterococcus* were pursued. Species were confirmed by either matrix-assisted laser desorption ionization–time of flight (MALDI-TOF) mass spectrometry or Biolog GenIII microplates using standard methods as described previously (2). A subset of 18 coisolated APEC/E. faecalis pairs was selected for whole-genome sequencing.

APEC and E. faecalis genomic DNA were isolated from overnight broth cultures grown for 12 to 14 h at 37°C in Luria or brain heart infusion broth, respectively. The DNeasy PowerLyzer microbial kit (Qiagen, Germantown, MD) was used for APEC DNA isolation, and the MasterPure Gram-positive DNA purification kit (Lucigen Corporation, Middleton, WI) was used for E. faecalis DNA isolation. DNA quality and quantity were assessed with a Qubit 4.0 fluorometer and the double-stranded (dsDNA) high-sensitivity assay kit (ThermoFisher Scientific, Waltham, MA). Pooled libraries with an insert size of ~350 bp were prepared with the Nextera DNA flex library prep kit (Illumina, San Diego, CA). Sequencing was completed on the Illumina MiSeq platform with paired-end 150-nucleotide reads and V2 chemistry using a MiSeq v2 reagent kit (500 cycles) according to the manufacturer’s instructions. Sequencing reads were assessed for quality using the MicroRunQC version 3 workflow within GalaxyTrakr (4). Contigs with a minimum length of 200 bp were assembled *de novo* and arranged into scaffolds in CLC Genomics Workbench version 22.0.1 (Qiagen). All sequences were annotated using the National Center for Biotechnology Information (NCBI) Prokaryotic Genome Automatic Annotation Pipeline version 6.3 (PGAAP) (5). Default parameters were used for all software unless otherwise specified. Genome coverage ranged from 42.4 to 107.8× (average, 63.75×) for APEC and 66.8 to 329.3× (average, 136.1×) for E. faecalis. Contig *N*_50_ values ranged from 43,161 to 368,859 bp (average, 142,982 bp) for APEC and 56,782 to 1,499,542 bp (average, 277,112 bp) for E. faecalis. Isolate details, sequencing and assembly statistics, and GenBank accession numbers for raw reads and draft genome assemblies are presented in Table 1.

**TABLE 1:** NCBI accession numbers and assembly metrics of Escherichia coli and Enterococcus faecalis draft genomes^*a*^

Species	Coisolate pair source^*b*^	Strain	Alias^*c*^	SRA accession	No. of reads	No. of contigs^*d*^	Genome size (bp)^*e*^	G+C content (%)^*f*^	*N*_50_ contig size (bp)^*e*^	Genome coverage (×)^*e*^	No. of coding sequences^*e*^	Genome accession
E. coli	1 _Chicken spleen_	NC_LB-3496	EC_01SN	SRS12250365	706,376	123	4,977,252	50.6	96,688	58.7	4,860	ABFMQN000000000
E. coli	2 _Chicken spleens_	NC_LB-3498	EC_02SN	SRS12250366	674,854	90	4,765,472	50.8	110,053	57.5	4,430	ABFJHU000000000
E. coli	3 _Turkey yolk sacs_	NC_LB-4777	EC_03YS	SRS2627921	716,379	173	5,059,298	50.5	190,604	54.9	4,716	ABDAWG000000000
E. coli	4 _Turkey yolk sacs_	NC_LB-4781	EC_04YS	SRS2627217	743,388	109	4,961,207	50.6	184,600	58.9	4,612	AASIBD000000000
E. coli	6 _Chicken yolk sacs_	NC_LB-5134	EC_06YS	SRS12250369	829,459	100	5,170,844	50.7	85,947	65.1	4,740	ABFMQO000000000
E. coli	8 _Chicken yolk sacs_	NC_LB-5139	EC_08YS	SRS12250368	840,753	157	5,135,900	50.6	78,336	67.3	4,841	ABFJVX000000000
E. coli	9 _Chicken yolk sacs_	NC_LB-5141	EC_09YS	SRS12250370	718,031	137	5,051,849	50.6	77,087	58.3	4,742	ABFJHV000000000
E. coli	10 _Chicken yolk sacs_	LB_5144	EC_10YS	SRS6919698	1,308,382	60	5,061,234	50.5	180,385	107.8	4,672	ABCWWH000000000
E. coli	11 _Chicken yolk sacs_	NC_LB-5146	EC_11YS	SRS12250371	769,195	80	4,912,666	50.6	111,173	63.7	4,455	ABFJHW000000000
E. coli	12 _Chicken yolk sacs_	NC_LB-5148	EC_12YS	SRS12250372	806,407	200	4,641,806	50.9	43,161	75.7	4,397	ABFMQR000000000
E. coli	13 _Chicken yolk sacs_	NC_LB-5151	EC_13YS	SRS12250374	800,055	107	5,237,141	50.5	110,128	64.5	4,868	ABFMQP000000000
E. coli	16 _Chicken yolk sacs_	NC_LB-5157	EC_16YS	SRS12250373	659,945	139	5,038,241	50.6	72,976	51.2	4,723	ABFMQU000000000
E. coli	21 _Chicken yolk sacs_	NC_LB-5172	EC_21YS	SRS12250375	846,593	59	4,963,053	50.5	161,311	70.9	4,522	ABFMQT000000000
E. coli	30 _Turkey yolk sacs_	LB_3186	EC_30YS	SRS6919697	1,316,742	258	5,268,314	50.5	195,336	102.7	4,987	AAUEYY000000000
E. coli	33 _Turkey yolk sacs_	LB_4788	EC_33YS	SRS6919707	546,675	191	5,199,460	50.7	130,135	42.4	4,850	AAUEYN000000000
E. coli	34 _Turkey yolk sacs_	LB_4801	EC_34YS	SRS6919846	1,069,610	102	5,074,369	50.7	173,959	51.5	4,763	AAUEYM000000000
E. coli	44 _Chicken yolk sacs_	LB_5326	EC_44YS	SRS4661859	614,811	92	4,825,025	50.7	368,859	53.1	4,542	AASEII000000000
E. coli	45 _Chicken yolk sacs_	LB_5328	EC_45YS	SRS4661863	553,644	131	5,345,637	50.5	202,944	43.3	4,984	AASEIN000000000
E. faecalis	1 _Chicken spleen_	NC_Ent1	EF_01SN	SRS13545784	622,502	70	2,940,645	37.3	94,997	90.4	2,777	JAOBYC000000000
E. faecalis	2 _Chicken spleens_	NC_Ent2	EF_02SN	SRS13545785	481,822	46	2,792,953	37.5	101,812	73.1	2,602	JAOBYB000000000
E. faecalis	3 _Turkey yolk sacs_	NC_LB-4778	EF_03YS	SRS13147985	2,331,246	262	2,956,381	37.5	379,473	329.3	2,844	JAOBYA000000000
E. faecalis	4 _Turkey yolk sacs_	NC_LB-4782	EF_04YS	SRS13147987	1,072,696	76	2,961,277	37.3	337,304	146.1	2,750	JAOBXZ000000000
E. faecalis	6 _Chicken yolk sacs_	NC_Ent6	EF_06YS	SRS13545786	566,707	71	2,901,603	37.4	56,782	83.1	2,732	JAOBXY000000000
E. faecalis	8 _Chicken yolk sacs_	NC_Ent8	EF_08YS	SRS13545787	649,240	44	2,909,376	37.4	132,820	94.1	2,751	JAOBXX000000000
E. faecalis	9 _Chicken yolk sacs_	NC_LB-5140	EF_09YS	SRS13147989	1,068,462	46	3,047,738	37.2	143,246	142.9	2,886	JAOBXW000000000
E. faecalis	10 _Chicken yolk sacs_	NC_LB-5143	EF_10YS	SRS13147991	1,053,136	216	3,254,584	37	327,347	133.8	3,087	JAOBXV000000000
E. faecalis	11 _Chicken yolk sacs_	NC_Ent11	EF_11YS	SRS13545788	758,911	49	3,025,734	37.2	131,206	107.5	2,840	JAOBXU000000000
E. faecalis	12 _Chicken yolk sacs_	NC_Ent12	EF_12YS	SRS13545789	625,895	62	3,005,661	37.3	141,703	89	2,826	JAOBXT000000000
E. faecalis	13 _Chicken yolk sacs_	NC_Ent13	EF_13YS	SRS13545790	474,903	39	3,032,509	37.2	154,252	66.8	2,838	JAOBXS000000000
E. faecalis	16 _Chicken yolk sacs_	NC_Ent16	EF_16YS	SRS13545791	618,879	56	2,908,728	37.5	116,454	90.4	2,702	JAOBXR000000000
E. faecalis	21 _Chicken yolk sacs_	NC_Ent21	EF_21YS	SRS13545793	646,983	96	2,841,247	37.5	71,864	97.3	2,718	JAOBXQ000000000
E. faecalis	30 _Turkey yolk sacs_	NC_LB-3185	EF_30YS	SRS13147992	1,332,622	142	2,957,175	37.5	201,937	171	2,816	JAOBXP000000000
E. faecalis	33 _Turkey yolk sacs_	NC_LB-4790	EF_33YS	SRS13147993	1,306,004	91	2,963,559	37.3	1,499,542	178.9	2,746	JAOBXO000000000
E. faecalis	34 _Turkey yolk sacs_	NC_LB-4803	EF_34YS	SRS13147995	1,601,810	186	2,993,152	37.4	647,371	223.3	2,812	JAOBXN000000000
E. faecalis	44 _Chicken yolk sacs_	NC_LB-5327	EF_44YS	SRS13147994	1,522,527	171	3,232,034	36.9	231,161	199.5	3,047	JAOBXM000000000
E. faecalis	45 _Chicken yolk sacs_	NC_LB-5329	EF_45YS	SRS13147998	926,196	30	2,903,886	37.3	218,748	132.7	2,712	JAOBXL000000000

a Genomes were coisolated from polymicrobial infections of chickens and turkeys at the North Carolina State University Veterinary Teaching Hospital in Raleigh, NC.

b E. coli and E. faecalis strains with common coisolate pair numbers were the only two species isolated from aerobic cultures of lesion swabs from chickens and turkeys.

c Strains were first named and described by Walker et al. (2) and renamed prior to sequencing.

d Contigs were split into scaffolds prior to annotation.

e Determined by the National Center for Biotechnology Information (NCBI) Prokaryotic Genome Automatic Annotation Pipeline (PGAAP) (5).

f Determined by CLC Genomics (Qiagen, Germantown, MD).

### Data availability.

The draft genome assemblies have been deposited in GenBank under BioProject accession numbers PRJNA293225 (Escherichia coli) and PRJNA837978 (Enterococcus faecalis). The versions described in this announcement are the second versions. Raw read files and genome assemblies can be accessed with the Sequence Read Archive (SRA) and genome accession numbers in Table 1, respectively.
